# Local mechanisms for loud sound-enhanced aminoglycoside entry into outer hair cells

**DOI:** 10.3389/fncel.2015.00130

**Published:** 2015-04-14

**Authors:** Hongzhe Li, Allan Kachelmeier, David N. Furness, Peter S. Steyger

**Affiliations:** ^1^Oregon Hearing Research Center, Oregon Health & Science UniversityPortland, OR, USA; ^2^School of Life Sciences, Keele UniversityStaffordshire, UK

**Keywords:** aminoglycoside, GTTR, outer hair cells, ototoxicity, auditory function, cellular uptake

## Abstract

Loud sound exposure exacerbates aminoglycoside ototoxicity, increasing the risk of permanent hearing loss and degrading the quality of life in affected individuals. We previously reported that loud sound exposure induces temporary threshold shifts (TTS) and enhances uptake of aminoglycosides, like gentamicin, by cochlear outer hair cells (OHCs). Here, we explore mechanisms by which loud sound exposure and TTS could increase aminoglycoside uptake by OHCs that may underlie this form of ototoxic synergy. Mice were exposed to loud sound levels to induce TTS, and received fluorescently-tagged gentamicin (GTTR) for 30 min prior to fixation. The degree of TTS was assessed by comparing auditory brainstem responses (ABRs) before and after loud sound exposure. The number of tip links, which gate the GTTR-permeant mechanoelectrical transducer (MET) channels, was determined in OHC bundles, with or without exposure to loud sound, using scanning electron microscopy. We found wide-band noise (WBN) levels that induce TTS also enhance OHC uptake of GTTR compared to OHCs in control cochleae. In cochlear regions with TTS, the increase in OHC uptake of GTTR was significantly greater than in adjacent pillar cells. In control mice, we identified stereociliary tip links at ~50% of potential positions in OHC bundles. However, the number of OHC tip links was significantly reduced in mice that received WBN at levels capable of inducing TTS. These data suggest that GTTR uptake by OHCs during TTS occurs by increased permeation of surviving, mechanically-gated MET channels, and/or non-MET aminoglycoside-permeant channels activated following loud sound exposure. Loss of tip links would hyperpolarize hair cells and potentially increase drug uptake via aminoglycoside-permeant channels expressed by hair cells. The effect of TTS on aminoglycoside-permeant channel kinetics will shed new light on the mechanisms of loud sound-enhanced aminoglycoside uptake, and consequently on ototoxic synergy.

## Introduction

Aminoglycoside antibiotics, like gentamicin and tobramycin, are clinically-essential antibiotics for treating life-threatening Gram-negative bacterial infections in premature babies or patients with cystic fibrosis, Gram-positive infections like tuberculosis, and protozoal infections (Durante-Mangoni et al., 2009; Zimmerman and Lahav, 2013). Despite their wide use, broad-spectrum bactericidal efficacy, and low cost, clinical dosing with aminoglycosides is limited by the risk of acute kidney damage and life-long hearing loss, with significant ramifications for quality of life (Forge and Schacht, 2000; Zimmerman and Lahav, 2013).

In the mammalian cochlea, auditory sensory cells, particularly outer hair cells (OHCs), are more susceptible to aminoglycoside-induced cytotoxicity than other cochlear cells, particularly at the base of the cochlea most sensitive to high frequency sound. Once these sensory cells are lost, they cannot be regenerated, leading to life-long hearing loss and deafness. Thus, extensive efforts are underway to ameliorate and prevent aminoglycoside-induced hair cell death.

Aminoglycosides can rapidly cross the blood-labyrinth barrier (BLB) into cochlear tissues and fluids (Tran Ba Huy et al., 1986; Li and Steyger, 2011), and enter hair cells through a number of routes. The best-characterized route is permeation through the mechanoelectrical transduction (MET) channel (Marcotti et al., 2005; Alharazneh et al., 2011). The MET channel is mechanically-gated by extracellular, heterodimeric tip links between adjacent stereocilia (Kazmierczak et al., 2007). Other mechanisms by which aminoglycosides can enter hair cells include endocytosis (Hashino and Shero, 1995), and permeation through other aminoglycoside-permeant cation channels expressed by hair cells, such as TRPV4 on apical membranes (Karasawa et al., 2008), or TRPA1 on basolateral membranes, of OHCs (Stepanyan et al., 2011).

*In vitro* studies show that when the MET channel is blocked, there is significantly reduced entry of aminoglycosides, or other organic cations such as FM1–43, into hair cells and reduced hair cell death (Gale et al., 2001; Marcotti et al., 2005; Wang and Steyger, 2009; Alharazneh et al., 2011), suggesting that the MET channel is a primary conduit for aminoglycoside entry into hair cells. Additionally, *in vitro* studies demonstrate that when tip links are broken or congenitally absent, hair cells show reduced uptake of aminoglycosides or FM1–43, and reduced drug-induced cytotoxicity (Seiler and Nicolson, 1999; Gale et al., 2001; Alharazneh et al., 2011; Vu et al., 2013). If extant tip links are broken, they can be regenerated within 24–48 h to restore MET function, and aminoglycoside uptake (Zhao et al., 1996; Vu et al., 2013). Tip link regeneration may represent one mechanism underlying temporary threshold shifts (TTS) in auditory perception (Zhao et al., 1996) following exposure to brief periods of intense sound such as explosions, gunfire, or loud music at rock concerts. The physiological effects of TTS also include transient reduction of endolymphatic potential (EP; Syka et al., 1981) and hair cell depolarization via purinergic receptors (Housley et al., 2013). Loud sound exposure has long been known to exacerbate aminoglycoside-induced ototoxicity in rodent studies (Brown et al., 1978; Gannon et al., 1979). We previously demonstrated that aminoglycoside exposure during moderate-to-intense sound exposure increases murine hair-cell uptake of fluorescently-tagged gentamicin (GTTR), elevating the risk of ototoxicity (Li et al., 2011). Experimentally, loud sound levels break tip links *in vivo* and close mechanically-gated MET channels (Husbands et al., 1999; Kurian et al., 2003; Sellick, 2007; Sellick et al., 2007) would be expected to reduce OHC uptake of aminoglycosides. This raises the question of how loud sound-enhanced GTTR uptake by OHCs occurs. To answer this question we conducted experiments to correlate the degree of tip link breakage in OHCs with uptake of GTTR. We found that loud sound-induced TTS was accompanied by a significantly decreased percentage of surviving OHC stereociliary tip links, while increasing OHC uptake of GTTR. This finding suggests that the tip link-gated MET channel is not the only conduit for GTTR entry into hair cells. We suggest several potential routes of entry for GTTR into hair cells in the presence of noise damage including changes in the MET channel following tip-link loss, endocytosis and basolateral channels, and further experimental hypotheses that if tested will shed new light on the mechanisms of loud sound-enhanced aminoglycoside uptake, and the ototoxic synergy between sound and aminoglycosides.

## Material and Methods

### Mice

To induce TTS, adult C57Bl/6 mice at 6–7 weeks of age with positive Preyer’s reflex were exposed to wideband noise (WBN; 86, 91 or 96 dB SPL) for 6 h/day for 3 days (Li et al., 2011). Mice were then intra-peritoneally (*i.p*.) injected with fluorescently-conjugated gentamicin (GTTR, 2 mg/kg) dissolved in phosphate buffered saline (PBS, pH = 7.4) within 30 min of cessation of noise exposure on the 3rd day, to track gentamicin uptake as previously described (Li et al., 2011). Control animals without noise exposure also received GTTR. Thirty minutes after GTTR administration, all mice were deeply anesthetized, and cardiac-perfused with PBS, then 4% formaldehyde. Fixed cochlear tissues were excised and processed for confocal microscopy and fluorescence quantification (Wang and Steyger, 2009). The care and use of all animals reported in this study was approved by the Animal Care and Use Committee of Oregon Health and Science University (IACUC approval #IS00001801).

### TTS Assessment

**Figure 1:**
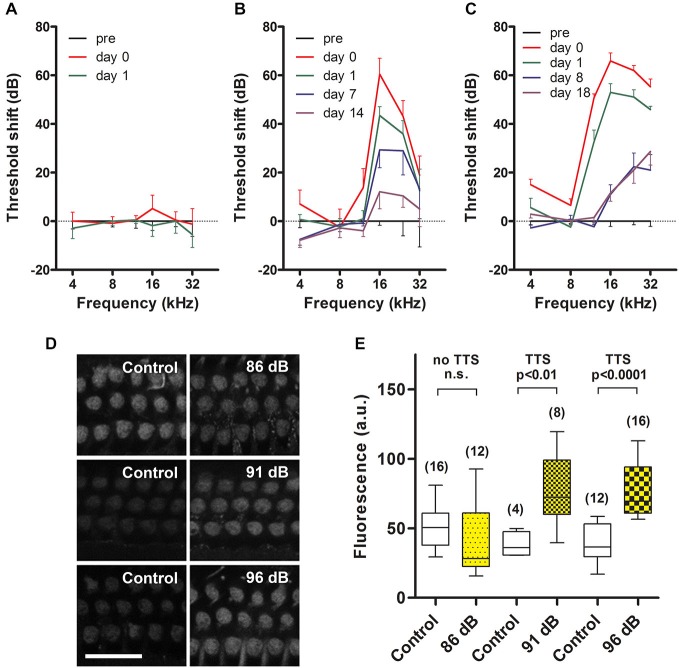
**Temporary threshold shifts (TTS) and sound-enhanced GTTR uptake by OHCs. (A)** Prolonged wide-band noise (WBN) at 86 dB SPL for 18 h over 3 days did not induce TTS (*n* = 3 mice/6 ears tested), nor enhance GTTR uptake by OHCs from the mid-basal coil of the cochlea (**D**, top row). **(B, C)** Prolonged WBN at 91 and 96 dB SPL for 18 h over 3 days did induce TTS that was maximal immediately after sound exposure, and mostly recovered over the next two weeks. Note that some permanent threshold shifts were observed at 16, 24 and 32 kHz 2 weeks after exposure to 91 dB SPL (*n* = 3/6 ears), and were more protracted 18 days after 96 dB SPL (*n* = 4/8 ears). Sound-induced TTS was also manifested in a broader frequency range at 96 dB SPL (12–32 kHz in **C**) compared to 91 dB SPL (16–32 kHz in **B**). **(D)** TTS induced by 91 and 96 dB SPL WBN enhanced GTTR uptake by OHCs from the mid-basal coil of the cochlea (middle and bottom rows). Note that experiments at different levels of sound exposure were conducted independently of each other, thus GTTR uptake was only comparable within experiments, i.e., horizontally within rows in **(D)**. Scale bar = 25 µm. **(E)** Sound-enhanced uptake of GTTR was significantly elevated after exposure to 91 (*n* = 2 for control, 4 for sound exposure) and 96 dB SPL (*n* = 3 for control, 4 for sound exposure), using unpaired *t* test. GTTR uptake was not further elevated at 96 dB SPL in the mid-basal cochlear regions. For 86 dB SPL comparison, *n* = 2 for control, 4 for sound exposure. Box represents the 25 and 75 percentiles of each dataset, while whiskers represent the minimum and maximum of each dataset. Number with parenthesis above the whiskers is the number of images analyzed per experimental condition. n.s. = not significant.

Mice were assessed by auditory brainstem response (ABR) measurements to determine the degree of TTS and time course of hearing recovery. Briefly, ABRs to 1 ms rise-time tone burst stimuli at 4, 8, 12, 16, 24 and 32 kHz, with 5 dB steps, were recorded and thresholds obtained for each ear, using an in-house system described previously (Mitchell et al., 1996). ABRs were obtained from both ears of each mouse the day before loud sound exposure (denoted as “pre” in Figures 1A–C), within 1 h (“day 0”), as well as one day (“day 1”), one week and two weeks after loud sound exposure was completed. Prior to ABR recording, mice were anesthetized *i.p*. with ketamine (65 mg/kg) and xylazine (13 mg/kg) and placed on a heating pad in a sound-proof, electrically isolated chamber. Needle electrodes were placed subcutaneously below the test ear, at the vertex, and with a ground on the claw. Each ear was stimulated separately with a closed tube sound delivery system sealed into the ear canal. ABR threshold was defined as the sound level at which the minimum identifiable ABR response occurred.

### Imaging and Data Analysis

The intensity of GTTR fluorescence was measured in single plane images of OHCs and pillar cells obtained with confocal settings kept identical across experiments, and processed in Photoshop and FIJI (ImageJ) as described previously (Wang and Steyger, 2009). An additional cohort of mice, exposed to 91 dB SPL WBN, was cardiac-perfused with 2.5% glutaraldehyde in 0.1 M sodium cacodylate buffer, and cochlear tissues processed for field emission scanning electron microscopy (FESEM) using the osmium-thiocarbohydrazide impregnation method (Furness and Hackney, 1986). The number of stereociliary tips and visible tip links per hair bundle (10 bundles per OHC row) was counted at a mid-cochlear location corresponding to a frequency range of 12–16 kHz. Student’s *t*-test and 1-way ANOVA with Tukey’s *post hoc* comparisons test were used to determine any significant differences between treatment groups.

## Results

### Enhanced GTTR Uptake by OHCs in Cochlear Regions with TTS

We systematically measured the degree of TTS induced by 3 different sound levels and assessed ABR threshold at multiple time points after sound exposure. This allowed us to assess the degree of TTS and correlate the degree of TTS and GTTR uptake for each sound protocol. Since serial ABR measurements are required to determine the degree of TTS, an independent set of mice was examined to determine GTTR uptake for each sound protocol.

**Figure 2:**
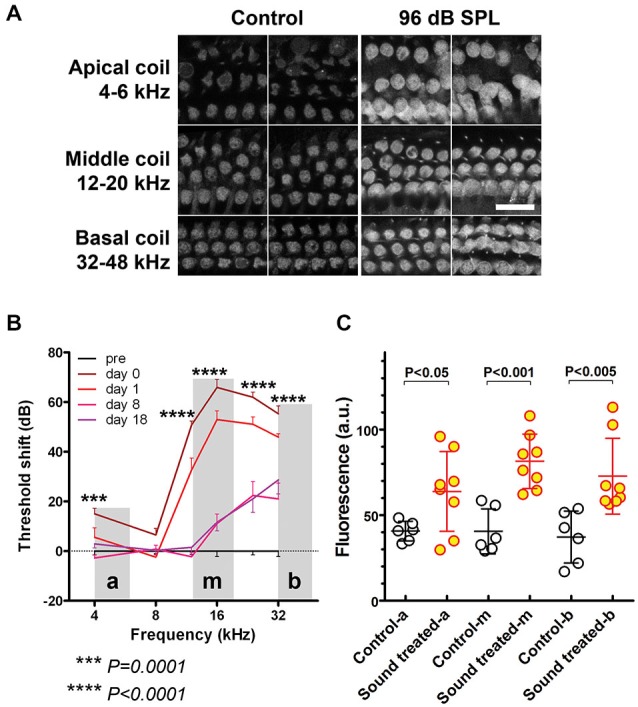
**Regions with sound-enhanced GTTR uptake correspond to regions with TTS. (A)** GTTR fluorescence in OHCs from apical, middle, and basal coils, representing 4–6 kHz, 12–20 kHz, and 32–48 kHz respectively, determined by the murine frequency-place map ImageJ plug-in.^1^ Scale bar = 25 µm. OHCs in loud sound-treated cochleae exhibited greater GTTR fluorescence compared to OHCs in all coils from control cochleae. **(B)** 96 dB SPL WBN resulted in a high-pass shaped TTS curve, minimal, yet significant, TTS at lower frequencies, i.e., 4–8 kHz, and TTS up to 70 dB at higher frequency regions, i.e., 12–32 kHz, using unpaired *t*-tests. “**a**”, “**m**”, and “**b**” depict apical, middle and basal cochlear regions respectively. Shaded areas depict cochlear regions where GTTR fluorescence was imaged and quantified. **(C)** WBN-induced sound-enhanced GTTR uptake is statistically significant at all examined frequency regions, using unpaired *t*-tests. Mean and s.d. are superimposed on each dataset.

Prolonged wide-band noise (WBN) at 86 dB SPL for 18 h over 3 days did not induce TTS (Figure 1A). Prolonged WBN at 91 and 96 dB SPL did induce TTS (Figures 1B,C). Elevation in sound-induced TTS (>50 dB) was more prevalent over a broader frequency range after 96 dB SPL sound treatment (12–32 kHz, Figure 1C) than at 91 dB sound exposure (16–32 kHz, Figure 1B). Exposure to 96 dB SPL WBN also induced a smaller, yet significant, TTS (<20 dB) in the lower 4-kHz frequency region (Figure 2B) that was not evident after exposure at 91 dB SPL. This smaller TTS in the 4-kHz region after 96 dB SPL exposure (and also at the 12-kHz region after 91 dB SPL exposure) recovered quickly within 24 h. Recovery from major TTS, 12 kHz and above after 96 dB SPL exposure and 16 kHz and above after 91 dB SPL exposure, was longer, and recovery took up to 2 weeks, and some permanent threshold shifts were seen at 24 and 32 kHz after 96 dB SPL exposure.

Exposure to prolonged WBN at 86 dB SPL which did not induce TTS (Figure 1A) did not enhance GTTR uptake by OHCs in the mid-basal coil of the cochlea (Figure 1D). Prolonged exposure to WBN at 91 and 96 dB SPL that did induce TTS (Figures 1B,C) also enhanced GTTR uptake by OHCs in the mid-basal coil of the cochlea (Figure 1D). However, loud (91 dB SPL) sound-enhanced OHC uptake of GTTR in the mid-basal coil of the cochlea appeared not further increased after exposure to higher sound levels (96 dB SPL; Figure 1E).

### Regions with Enhanced GTTR Uptake are Broader than Regions with Major Elevations in TTS

All OHCs in cochleae with 96 dB SPL-induced TTS exhibited greater GTTR fluorescence compared to OHCs from equivalent regions in control cochleae (Figure 2A). GTTR fluorescence intensity was assessed in OHCs from apical, middle, and basal coils, representing a frequency range of 4–6 kHz, 12–20 kHz, and 32–48 kHz respectively, as determined by a murine frequency-location map (Ou et al., 2000; Müller et al., 2005). This enabled comparison of the degree of enhanced GTTR uptake at various cochlear tonotopic locations. In apical, 4-kHz frequency cochlear regions, 96 dB SPL WBN induced a small TTS that was generally less than 20 dB (Figure 2B), but significantly enhanced OHC uptake of GTTR compared to OHCs from equivalent regions in control cochleae (Figure 2C). At basal, 12–32 kHz, cochlear regions, 96 dB SPL WBN induced threshold shifts up to 70 dB, and more statistically significant OHC uptake of GTTR than in OHCs from equivalent regions in control cochleae (Figures 2B,C).

### Sound-Enhanced GTTR Uptake is More Robust in OHCs than in Pillar Cells

**Figure 3:**
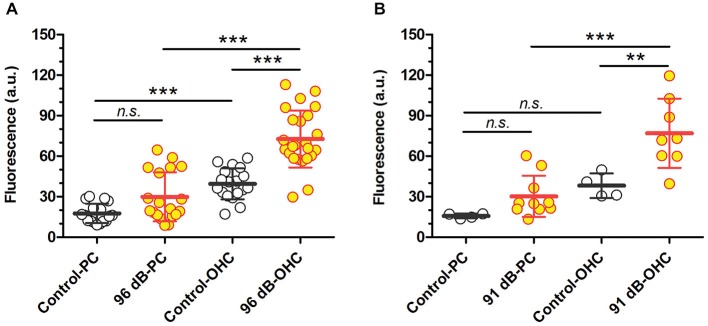
**Loud sound-enhanced GTTR uptake in two cell populations from the organ of Corti after 96 dB SPL (A) or 91 dB SPL (B) sound exposure. (A)** Sound-enhanced GTTR fluorescence was observed in a fraction of pillar cells from the middle and basal cochlear regions of 2 out of 4 cochleae after exposure to WBN at 96 dB SPL for 18 h over 3 days. In contrast, enhanced GTTR fluorescence was observed in the majority of OHCs adjacent to pillar cells in the same regions. *n* = 3 for control cochleae, *n* = 4 for loud sound-treated cochleae; up to six sites were examined for each cochlea and averaged from at least 16 pillar cells or OHCs. **(B)** Sound-enhanced GTTR fluorescence was observed in a few pillar cells from the middle cochlear regions after exposure to WBN at 91 dB SPL for 18 h over 3 days. In contrast, enhanced GTTR fluorescence was observed in the majority of OHCs adjacent to pillar cells in the same regions. *n* = 2 for control cochleae, *n* = 4 for sound-treated cochleae; up to six sites examined for each cochlea and averaged from at least 9 pillar cells or OHCs. For both sound levels, sound-enhanced GTTR uptake is only statistically significant in OHC populations, using Tukey’s multiple comparison test from one-way ANOVA. ***p < 0.001, **p < 0.01, *n.s*. = not significant. Mean and s.d. are superimposed on each dataset.

Previously we demonstrated that TTS increased cochlear uptake of GTTR, particularly in marginal cells adjacent to vasodilated capillaries in the *stria vascularis* (Li et al., 2011), which was also observed here (data not shown). In this experiment, we assessed whether major TTS is accompanied by enhanced uptake of GTTR in pillar cells (as a proxy for all supporting cells within the organ of Corti), or preferentially by OHCs, by comparing the relative increases in GTTR fluorescence after induction of TTS in OHCs and adjacent pillar cells. After exposure to prolonged WBN at 96 dB SPL, only a third of pillar cells displayed enhanced GTTR uptake, yet the majority of OHCs in the same region displayed enhanced GTTR uptake (Figure 3A; Table 1). A one-way ANOVA with *post hoc* Tukey’s multiple comparison test revealed significant increases in GTTR fluorescence in OHCs, but not pillar cells, after loud sound exposure (Table 1). Data obtained from cochleae after prolonged sound exposure to 91 dB SPL presented a similar trend (Figure 3B; Table 1). This suggests that OHCs appear to possess at least one mechanism that facilitates greater GTTR uptake compared to pillar cells.

**Table 1:** **Loud sound exposure induced greater GTTR uptake by OHCs compared to pillar cells**.

		96 dB SPL			91 dB SPL*		
		*N*	Mean ± s.e.m.	Tukey’s test#	*N*	Mean ± s.e.m.	Tukey’s test#
**OHCs**	**Control**	18	39.49 ± 2.696	*p* < 0.001	4	38.13 ± 4.541	*p* < 0.001
	**Sound-treated**	24	72.70 ± 4.306		8	76.96 ± 9.068
**Pillar cells**	**Control**	17	17.59 ± 1.740	*p* > 0.05 (n.s.)	4	15.70 ± 0.8995	*p* > 0.05 (n.s.)
	**Sound-treated**	19	29.87 ± 4.173		10	30.20 ± 4.803

*Only cells from the middle coil of 91 dB SPL sound-exposed cochleae (and their respective controls) were imaged, while cells from apical, middle and basal cochlear regions of 96 dB SPL sound-exposed cochleae (and their respective controls) were imaged and GTTR fluorescence quantified (arbitrary unit). #Tukey’s multiple comparison test from one-way ANOVA including all four conditions: Control-OHC, Sound-treated-OHC, Control-PC and Sound-treated-PC. p < 0.05 is significant. PC = Pillar cells. N = number of images, multiple cells per image were averaged.

### Noise Exposure Reduces the Number of Tip Links

**Figure 4:**
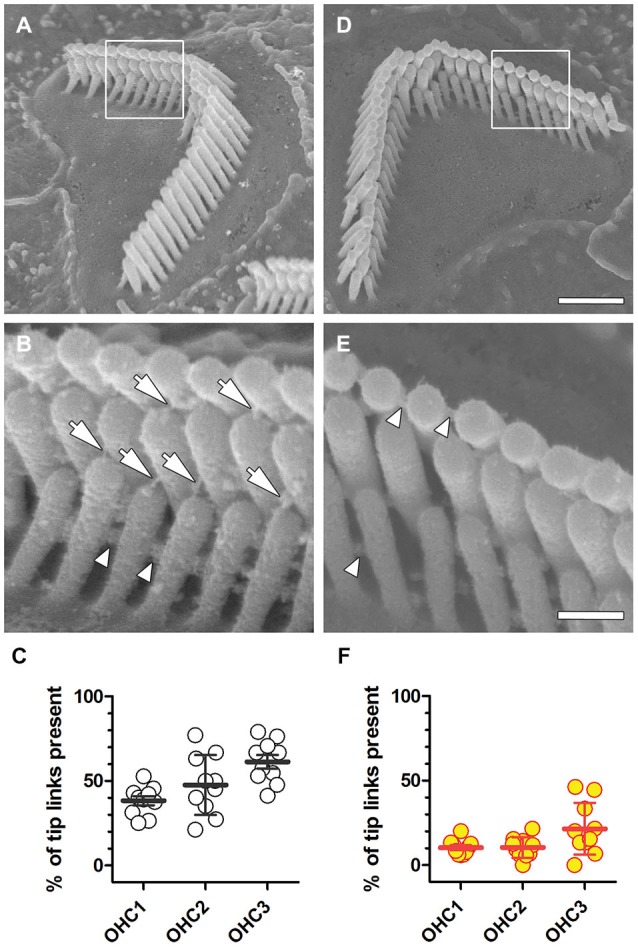
**Reduced tip-link survival after noise exposure. (A)** A representative stereociliary bundle from the second OHC row in the middle coil of a control cochlea. Framed area is enlarged in **(B)**, arrows indicate identifiable tip links, and arrowheads point to identifiable side links. **(C)** The percentage of tip-links in OHC rows from the middle coil in a representative control mouse that survived processing for SEM is shown. Each symbol represents the percentage of tip-links observed in an individual OHC bundle (*n* = 10 per row), with mean and s.d. superimposed. **(D)** A representative stereociliary bundle from the third OHC row in the middle cochlear coil of a representative mouse exposed to WBN at 91 dB SPL for 18 h over 3 days displaying normal gross bundle morphology, but no visible tip links **(E)**, while lateral links between stereocilia of the same row are still present. **(F)** Following prolonged WBN at 91 dB SPL for 18 h, the percentage of tip-links surviving sound exposure and SEM processing are substantially reduced in all three OHC rows. Scale bar = 1.0 µm in **(D)** also applies to **(A)**. Scale bar = 250 nm in **(E)**, also applies to **(B)**.

Scanning electron microscopy of OHCs with or without prolonged exposure to WBN at 91 dB SPL, revealed OHCs with a classic “V” or “W” stereociliary bundle (Figures 4A,D). In OHCs without exposure to WBN, tip-links between stereocilia of adjacent rows were clearly identifiable (Figure 4B). The percentage of tip links present in representative OHCs in the middle cochlear coil of control mice varied between OHC rows, ranging from 38% for the innermost row to 61% for the outermost row (Figure 4C; Table 2). Prolonged exposure to WBN at 91 dB SPL caused no obvious disarray of bundle morphology in OHCs from equivalent regions to those in control cochleae (Figure 4D). Nonetheless, substantially fewer tip links were observed in all three rows of OHCs in loud sound-exposed cochleae compared to OHCs from equivalent regions in control cochleae (Figures 4E,F; Table 2). This reduction in tip link survival was statistically significant for all three row-to-row comparisons using unpaired *t*-tests with Welch’s correction (*p* < 0.0001).

**Table 2:** **Tip link numbers decreased in OHCs after 91 dB SPL sound exposure**.

Control			91 dB SPL		
Animal ID	Row	Mean % ± s.e.m.	Animal ID	Row	Mean % ± s.e.m.
2	1	13.03 ± 3.541	5	1	3.061 ± 1.287
	2	42.04 ± 5.008		2	1.891 ± 0.9688
	3	44.97 ± 4.703		3	11.52 ± 1.673
	Overall	**33.35 ± 3.654**		Overall	**5.492 ± 1.092**
3	1	38.20 ± 2.720	6	1	10.25 ± 1.388
	2	47.51 ± 5.5		2	10.29 ± 1.921
	3	61.25 ± 3.947		3	21.41 ± 4.864
	Overall	**48.99 ± 2.951**		Overall	**13.98 ± 1.995**
4	1	38.46 ± 3.062	7	1	61.45 ± 6.571
	2	56.73 ± 3.150		2	47.88 ± 6.124
	3	68.12 ± 5.681		3	46.06 ± 6.046
	Overall	**54.44 ± 3.237**		Overall	**51.80 ± 3.709**

*Ten hair bundles in each OHC row from the mid-cochlear region (12–16 kHz region) of each cochlea were examined (3 mice per group). The percentage of tip link survival was calculated as the number of visible tip links divided by identifiable stereociliary tips (bar the tallest row of stereocilia) × 100*.

## Discussion

The present study has demonstrated that: (i) moderate-to-loud sound level exposures (91 and 96 dB WBN for 6 h per day over 3 consecutive days) that induce TTS also increase OHC uptake of ototoxic aminoglycosides (GTTR) *in vivo*; (ii) higher levels of sound exposure (96 dB SPL) increase the degree of TTS and widen the region of GTTR uptake; and (iii) tip link survival in the region of increased GTTR uptake is reduced significantly. In addition, pillar cell uptake of GTTR is not significantly increased after loud sound exposure, suggesting that endolymph levels are statistically unchanged, and that increased OHC uptake of GTTR after loud sound exposure is hair cell-specific. These findings are clinically relevant as they provide additional evidence for a synergistic ototoxicity of loud sound exposure and aminoglycoside treatment (Gannon et al., 1979; Ryan and Bone, 1982). These findings also have implications for patients treated with aminoglycosides and (prior) exposure to high noise levels (Li and Steyger, 2009; Zimmerman and Lahav, 2013).

The findings have two additional implications. The first is that loss and regeneration of stereociliary tip links may explain loud sound-induced TTS. In organ of Corti explants, stereociliary tip links are regenerated 24–48 h after ablation, restoring functional kinetics and aminoglycoside permeability to MET channels (Indzhykulian et al., 2013; Vu et al., 2013). If this capability also exists *in vivo*, it may contribute to the recovery of auditory function from TTS in the 4 kHz region after 96 dB SPL WBN exposure. To test this hypothesis, it will be necessary to test whether the extent of tip-link loss and regeneration correlates with the degree of TTS and recovery observed in this region. Experiments to verify this are ongoing.

Other mechanisms besides loss of tip links could contribute to the TTS, including synaptic mechanisms (Chen et al., 2012; Liu et al., 2013), or a reduction (followed by recovery) in the EP (Syka et al., 1981; Ahmad et al., 2003). We used C57BL/6 mice that are resistant to changes in the EP after acoustic overstimulation (Ohlemiller and Gagnon, 2007), which reduced the contribution of EP loss allowing us to better evaluate the role of the organ of Corti in TTS.

The second major implication of these data is that GTTR uptake is enhanced in regions associated with TTS and significant loss of tip links. At first sight, this result is surprising as tip-link destruction would be predicted to *reduce* GTTR uptake. Tip links are required for gating the MET channels, and aminoglycosides enter hair cells through these channels when open. The critical evidence is that mutant hair cells lacking Pcdh15, one of the two main molecular components of the tip link, show substantially reduced tip-link numbers, and fail to load with aminoglycosides (Alagramam et al., 2011). Moreover, hair cells with double-homozygous mutations of the putative MET channel proteins TMC1 and TMC2, but with intact tip links, also fail to take up gentamicin (Kawashima et al., 2011; Pan et al., 2013). The calcium-binding site within the MET channel pore modulates MET channel conductance (Pan et al., 2012) and permeation by aminoglycosides. Increasing extracellular [Ca^2+^] reduces aminoglycoside permeation of MET channels (Marcotti et al., 2005; Coffin et al., 2009; Wang and Steyger, 2009), and is manifested by greater hair cell survival (Coffin et al., 2009; Alharazneh et al., 2011). Conversely, lower [Ca^2+^] increases MET channel conductance and aminoglycoside permeation, promoting hair cell death (Marcotti et al., 2005; Coffin et al., 2009; Alharazneh et al., 2011). With the loss of hair bundle tip-links gating the GTTR-permeant MET channels, we discuss below potential mechanisms that could facilitate this increased uptake of GTTR by OHCs after loud sound exposure.

### Increased Driving Potential may Enhance GTTR Uptake through Fewer Active Channels

The loss of tip links will have complex physiological consequences on MET channel kinetics. The open probability of OHC MET channels is ~50% (Cody and Russell, 1987; Legan et al., 2005), any loss of tip links would reduce the gross standing current through the remaining tip link-gated MET channels, and increase the EP (Sellick et al., 2007). The effect would increase the driving force from endolymph into hair cells and increase the rate of GTTR entry into OHCs via aminoglycoside-permeant channels (Marcotti et al., 2005).

### MET Channels may Still Permit Aminoglycoside Entry in the Absence of Tip Links

Although the identity of the MET channel remains uncertain, experimental evidence suggests that a mechanosensitive ion channel remains in hair cells lacking tip links through which aminoglycosides may still enter the cell. Meyer et al. (1998) recorded MET currents in isolated OHCs before and after tip link ablation using BAPTA and elastase. Since these currents were blocked by MET channel blockers including aminoglycosides, amiloride, and gadolinium, the loss of tip links could result in a tonically open channel.

In more recent studies of mice lacking tip link proteins, anomalous MET currents have been recorded in which the channels displayed reverse polarity, becoming open for hair bundle displacements in the normally negative direction (Alagramam et al., 2011). Similar results were obtained in TMC1/TMC2 double mutants, in normal mouse hair cells exposed to BAPTA and allowed to recover for several minutes (Kim et al., 2013), and in hair cells in which the bundle was severely mechanically damaged (Marcotti et al., 2014).

Two explanations have been posited to explain these data. The first is that a mechanically sensitive channel, for example a precursor of the MET channel itself, is unmasked in the hair-cell apex in these cases (Marcotti et al., 2014), or, alternatively, that the original MET channels are redistributed to a different part of the hair bundle where they still operate mechanically but in an abnormal, reversed fashion (Barr-Gillespie and Nicolson, 2013). One possible explanation, therefore, for the increased OHC uptake of GTTR despite reduced numbers of tip links is that some form of aminoglycoside-permeant MET channel or precursor allows aminoglycoside entry. However, this explanation is not consistent with the absence of aminoglycoside entry in TMC1/TMC2 double mutants or tip-link protein mutants, as noted earlier. The contribution of such unmasked or redistributed MET channels to GTTR uptake may not be substantial.

### Other Routes of Aminoglycoside Entry

#### Basolateral Membrane Mechanisms

One candidate aminoglycoside-permeant cation channel residing in the basolateral membrane of OHCs is TRPA1 (Stepanyan et al., 2011). These channels are inflammatory, irritant, and oxidative stress sensors (Kwan et al., 2006) and, hence, could be activated following the stress of noise damage. TRPA1 channels have a pore diameter of 1.1 nm (Karashima et al., 2010), sufficient to allow permeation by aminoglycosides, and show agonist-induced dilation (to ~1.4 nm) and permeation by organic cations (Chen et al., 2009; Banke et al., 2010; Karashima et al., 2010). The TRPA1 agonist, 4-hydroxynonenal, is elevated after noise exposure and *in vitro* facilitates increased OHC uptake of GTTR (Yamashita et al., 2005; Stepanyan et al., 2011).

Another basolateral channel, the nicotinic acetylcholine receptor (nAChR), may provide another route for the sound-enhanced aminoglycoside uptake by OHCs (Luk et al., 2014). Efferent activation of nicotinic acetylcholine receptor (nAChR) channels induces a calcium influx that opens nearby Ca^2+^-sensitive K^+^ channels (Murthy et al., 2009; Lipovsek et al., 2012). Aminoglycosides block efferent activation of acetylcholine-induced Ca^2+^ and K^+^ currents *in vitro* (Blanchet et al., 2000; Seluakumaran et al., 2008). There are 3 potential mechanisms by which activated nAChR channels may modulate OHC uptake of aminoglycosides: (1) through nAChR itself from perilymph; (2) through nearby Ca^2+^-sensitive K^+^ channels, such as SK2 (Murthy et al., 2009) and BK channels (Hafidi et al., 2005; Yu et al., 2014), although this would be against the efflux direction of the native cation (K^+^) current; and (3) via hyperpolarization of OHCs by activating outward potassium current (Housley and Ashmore, 1991; Doi and Ohmori, 1993) that increases the driving force through apically-located aminoglycoside-permeant channels, including MET, as described above.

These mechanisms are dependent on aminoglycoside entry into the scala tympani, which has been demonstrated (Tran Ba Huy et al., 1986; Hirose et al., 2014). However, uptake of aminoglycosides by healthy, physiologically-functioning OHCs from the scala tympani is far slower than from systemically-delivered aminoglycosides via endolymph (Li and Steyger, 2011). Nonetheless, aminoglycoside uptake by OHCs from the perilymphatic scala tympani may represent a mechanism of synergistic ototoxicity by OHCs over longer time frames, or after damaging sound exposures (Stepanyan et al., 2011).

#### Endocytosis

Hair cells are also able to endocytose aminoglycosides into the apical cytoplasm (Hiel et al., 1993; Hashino et al., 1997; Alharazneh et al., 2011). However, concanavalin A or Dynosore, non-selective blockers of endocytosis, did not significantly alter the rate of GTTR uptake, nor protect hair cells against aminoglycoside cytotoxicity (Alharazneh et al., 2011). Although TTS could enhance endocytosis of aminoglycosides *in vivo* by hair cells, delipidation of cochlear tissues to unmask GTTR fluorescence will remove endosome-associated GTTR fluorescence, and alternative unmasking steps should be utilized to further test this hypothesis (Myrdal et al., 2005).

#### Summary

This study reveals one possible contributor to loud sound-induced TTS is the loss of tip links, for which tip link regeneration may underlie the functional recovery associated with TTS. Furthermore, this study shows increased OHC uptake of GTTR during TTS that may contribute to the synergistic ototoxicity of noise and aminoglycosides. Finally, the apparent discrepancy between tip link loss and enhanced GTTR uptake may be explained by the activation of one or more additional routes of aminoglycoside entry into hair cells during TTS.

## Conflict of Interest Statement

The authors declare that the research was conducted in the absence of any commercial or financial relationships that could be construed as a potential conflict of interest.

## Footnotes

^1^http://www.masseyeandear.org/research/otolaryngology/investigators/laboratories/eaton-peabody-laboratories/epl-histology-resources/imagej-plugin-for-cochlear-frequency-mapping-in-whole-mounts/
